# Comprehensive strategy of sonodynamic therapy and gas therapy on tumor treatment

**DOI:** 10.4103/mgr.MEDGASRES-D-24-00152

**Published:** 2026-01-06

**Authors:** Lilin Zhao, Yaqi Zhang, Qian Huang, Ting Zhang

**Affiliations:** Department of Ultrasound, The Affiliated Cancer Hospital of Nanjing Medical University, Jiangsu Cancer Hospital, Jiangsu Institute of Cancer Research, Nanjing, Jiangsu Province, China

**Keywords:** combination therapy, gas therapy, hypoxia modulation, immunotherapy, photodynamic therapy, precision oncology, reactive oxygen species, sonodynamic therapy, tumor microenvironment, ultrasound-responsive material

## Abstract

Sonodynamic therapy has emerged as a novel non-invasive treatment for cancer with limited single effects. To achieve optimal therapeutic efficacy, sonodynamic therapy frequently needs to be combined with other therapeutic strategies. By exploiting the biological properties of specific gas molecules, gas therapy is an emerging tumor treatment that exerts direct or indirect inhibitory effects on tumor cells. This review systematically examines the rationales, methodologies, and outcomes of sonodynamic therapy and gas therapy combinatorial strategies for malignant tumors. There is a synergistic effect between sonodynamic therapy and gas therapy in tumor treatment. The ultrasound-induced cavitation enhances tissue permeability for improved gas delivery, while gas molecules concurrently sensitize sonodynamic reactions and ameliorate tumor hypoxia. The interaction significantly enhances the therapeutic effect of tumors. Moreover, the combination of sonodynamic therapy with other therapeutic modalities can significantly enhance the anti-tumor efficacy, improve the therapeutic precision and safety, while improve the tumor microenvironment. This combined treatment strategy can also reduce side effects and has a broad clinical application perspective.

## Introduction

**Figure 1:**
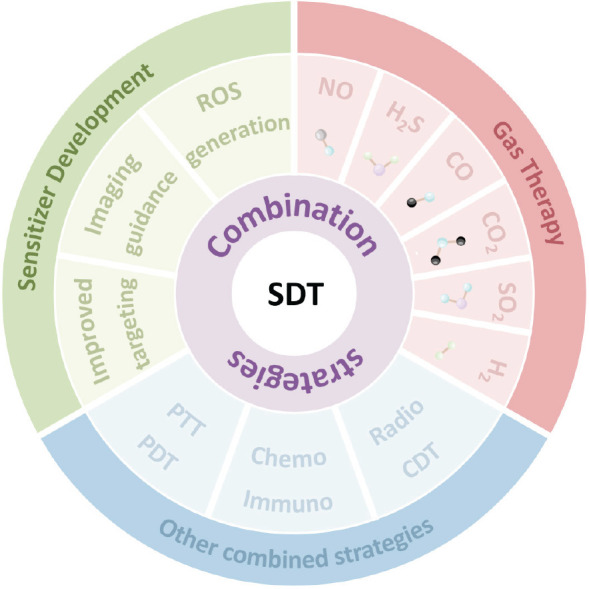
Sonodynamic-based combined therapeutic strategies. It illustrates the synergistic effects of SDT with various treatment modalities and covers the three main design directions of nanosonosensitizers. Created with Microsoft PowerPoint Office 2019. CDT: Chemodynamic therapy; Chemo: chemotherapy; CO: carbon monoxide; CO_2_: carbon dioxide; H_2_: hydrogen; H_2_S: hydrogen sulfide; Immuno: immunotherapy; NO: nitric oxide; PDT: photodynamic therapy; PTT: photothermal therapy; Radio: radiotherapy; ROS: reactive oxygen species; SDT: sonodynamic therapy; SO_2_: sulfur dioxide.

Tumor treatment methods are diverse, encompassing major approaches such as surgery, radiotherapy, chemotherapy, immunotherapy and targeted therapy.^1^,^2^ The aim is to inhibit or eliminate tumors by local or systemic means to prolong survival and possibly prevent further deterioration of the patient’s quality of life. In 1989, Yumita et al.^3^ observed that a sensitizer known as hematoporphyrin could effectively kill cancer cells in response to ultrasound, a process that was subsequently designated as sonodynamic therapy (SDT). Firstly, SDT has an effect on the cell membrane and causes a cavitation effect. The process of cavitation gives rise to the formation of microbubbles, which subsequently result in the formation of reversible or irreversible voids (acoustic pores) within the surrounding cells or on the endothelial cells of blood vessels.^4^ The utilization of ultrasound-induced alterations in cell membrane permeability has been demonstrated to enhance the delivery of drugs and genes into target cells, thereby augmenting the bioavailability of the therapeutic intervention.^5^ Secondly, SDT acts on the mitochondria of the tumor cells to exert a killing effect.^6^ Finally, SDT affects the structure and composition of tumor microenvironment (TME), thereby inhibiting the stock of tumor cells.^7^ In experimental models of animal tumors, SDT has exhibited considerable antitumor efficacy. The therapeutic benefits of SDT encompass not only the substantial reduction in tumor volume but also the inhibition of tumor metastasis and recurrence to a certain extent. The efficacy of individual SDT is restricted, and recent research has focused on enhancing the efficacy of SDT. Approaches to achieving this goal, the combination of SDT with chemotherapy, photodynamic and photothermal therapy, gas therapy, and immunotherapy will produce a synergistic effect (**Figure 1**). This review aimed to explore the experimental research and clinical translation of SDT in combination with gas therapy and other combination therapeutic modalities.

## Search Strategy

To achieve this objective, a comprehensive literature search was conducted via PubMed. The following terms were used: (“sonodynamic therapy” OR SDT) AND ((“gas therapy” OR “carbon monoxide” OR “nitric oxide”) OR (“combination therapy” OR “chemo-sonodynamic” OR “immuno-sonodynamic”)). The inclusion criteria were: (1) original research articles, systematic reviews, and meta-analyses that directly address the use of SDT in combination with gas therapy or other modalities. (2) Studies were published in English between the years 2019 and 2024. The exclusion criteria were articles not available in full text.

## Combined Strategy of Gas Therapy and Sonodynamic Therapy in Tumor Treatment

**Figure 2:**
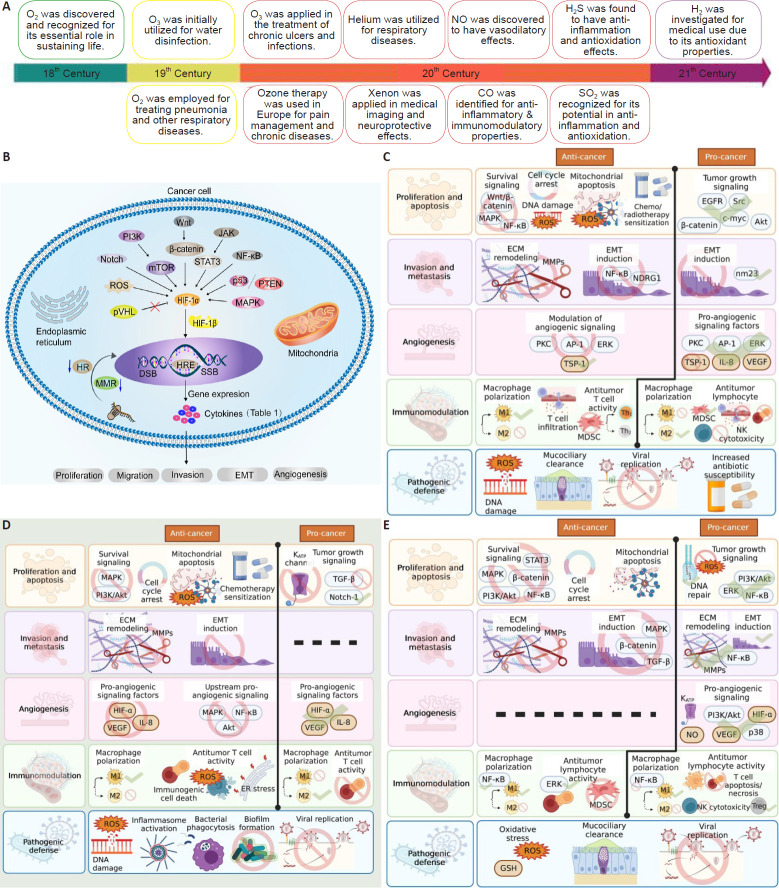
The history of gas therapy and the molecular mechanisms of major gases. (A) Advancements and key milestones in gas therapy. A was reprinted from Chen et al.^9^ (B) Biological changes in cancer cells adapt to hypoxia. B was reprinted from Chen et al.^10^ (C) NO effects in the context of cancer. (D) CO effects in the context of cancer. (E) H_2_S effects in the context of cancer. C–E were reprinted from Oza and Kashfi.^11^ Copyright 2023 Elsevier Inc. AP-1: Activator protein-1; CO: carbon monoxide; DSB: DNA double-strand break; ECM: extracellular matrix; EGFR: epidermal growth factor receptor; EMT: epithelial-mesenchymal transition; ERK: extracellular regulated protein kinases; H_2_: hydrogen; H_2_S: hydrogen sulfide; HIF: Hypoxia-inducible factor; HR: homologous recombination; HRE: hypoxia response element; IL: interleukin; JAK: Janus kinase; MAPK: mitogen-activated protein kinase; MDSC: Myeloid-derived suppressor cell; MMR: mismatch repair; mTOR: mammalian target of rapamycin; NO: nitric oxide; O_2_: oxygen; O_3_: ozone; PI3K: phosphatidylinositol 3-kinase; PKC: protein kinase C; PTEN: phosphatase and tensin homolog; pVHL: von hippel-landau; SO_2_: sulfur dioxide; SSB: single-stranded DNA-binding protein; STAT3: signal transducer and activator of transcription 3; TSP: total serum protein; VEGF: vascular endothelial growth factor.

Reactive oxygen species (ROS) production is dependent on sufficient oxygen levels; thus, the hypoxic microenvironment that characterizes most tumors can limit the efficacy of SDT. The use of gases to enhance SDT efficacy appears to be an applicable solution to this problem. Gas therapy has emerged as a promising therapeutic modality with applications ranging from antitumor to inflammatory and antioxidant therapies, thanks to its exploitation of biological effects exhibited by gas molecules (**Figure 2**).^8^,^9^,^10^,^11^ The application of aerodynamics in gas therapy offers novel approaches for enhancing the efficiency of drug delivery and potentiating its therapeutic efficacy. Beyond its involvement in the transport and distribution of gases within tissues, aerodynamics has been demonstrated to improve the precision and effectiveness of gas therapy by optimizing the penetration and release of gases in tumor tissues. For instance, by modulating the flow rate, pressure, and diffusion characteristics of gases, aerodynamics can enhance the uniform distribution of gas molecules within tumor tissues, thereby amplifying their biological effects.^12^ Moreover, the integration of aerodynamics with nanotechnology has the potential to engineer intelligent gas delivery systems, further enhancing the precision and controllability of gas therapy. The combination of SDT and gas therapy represents an emerging tumor treatment strategy that can enhance antitumor efficacy in multiple ways. At present, the principal gases used in the context of SDT combination therapy consist of nitric oxide (NO), carbon monoxide (CO), oxygen (O_2_), hydrogen sulfide (H_2_S), and sulfur dioxide (SO_2_).^13^ These gas molecules can perform a variety of functions, including the modulation of cell signaling, the induction of apoptosis, the inhibition of tumor growth, and the enhancement of TME. SDT, when coupled with gas therapy, exhibits a synergistic effect that operates through several mechanisms. On the one hand, the mechanical wave and thermal effect generated by SDT can increase the permeability of the tissue, thus promoting better penetration of the gas into the tumor tissue. On the other hand, the ROS produced by SDT can interact with bioactive molecules, thereby inducing a collaborative destruction effect. Additionally, this combination strategy has been demonstrated to modulate TME and optimize the immune response, in turn enhancing the therapeutic effect.^9^

### Nitric oxide

The mechanisms by which NO exerts its lethal effects on tumor cells are numerous.^14^ Firstly, NO itself could regulate the expression of various apoptotic genes and affect the mitochondrial respiratory chain, therefore inducing apoptosis in tumor cells. Secondly, NO can react with ROS to produce reactive nitrogen species, such as the highly reactive peroxynitrite (ONOO-), which can damage tumor cell DNA and modify mitochondrial membrane permeability.^15^ Inhibition of mitochondrial respiration has been found to alleviate the hypoxic microenvironment within the tumor, a prerequisite for NO to synergize with SDT. At present, the primary NO donors for SDT in conjunction with gas therapy are L-arginine (L-Arg) and nitroglutathione, as well as N-nitrosamines and metal nitroso complexes. L-Arg is the body’s natural donor of NO, and its biocompatibility makes it an optimal solution for delivering NO. Nie et al.^16^ reported a nanocatalyst platform, Arg@VMT@PDA-PEG, selecting L-Arg as a donor of NO, facilitating the use of chemodynamic therapy, SDT, and NO-based gas therapy for combined cancer treatment. Under ultrasound stimulation, activated oxygen not only performs SDT, but also oxidizes L-Arg to NO for gas therapy. Fluorescence staining revealed an overlap between areas of high ROS and ONOO-expression with areas of tumor damage, validating the synergistic effect of NO on SDT. The article further speculates that the reason NO enhances SDT may be related to the fact that NO has a longer lifespan and is able to diffuse freely from the interstitial space into the cell. In addition to the conventional L-Arg, An et al.^17^ selected nitroglutathione as the NO donor and devised a biomimetic nanoplatform GCZ@M with dual pH/ultrasound response for NO gas combined SDT treatment. *In vitro* and *in vivo* experiments indicated that the effect of SDT combined with gas therapy increased in a stepwise manner with the number of ultrasound, which may be attributable to the fact that the combination therapy alleviated the hypoxic microenvironment inside the tumor.

### Hydrogen sulfide

H_2_S functions as a gas signaling molecule, demonstrating inhibitory effects on tumor growth. This is thought to be related to several mechanisms, including the modulation of the microenvironmental hypoxia state and the impact on vascular endothelial permeability.^18^ H_2_S can also drive the iron death process of tumor cells by regulating homocysteine metabolism. These mechanisms underscore the use of H_2_S in gas therapy for tumors. Endogenous H_2_S is synthesized using L-cysteine (L-Cys) as a substrate, and based on this principle, Kuang et al.^19^ designed hydrogel nanocomplexes (CSSH-Gel) for the treatment of glioma. In the presence of ultrasound, L-Cys generates H_2_S, while the manganese porphyrin-based metal-organic framework produces 1O_2_. Consequently, the membrane potential of mitochondria declines in response to H_2_S and 1O_2_, resulting in mitochondrial dysfunction. Moreover, the study also demonstrated that the sustained release of H_2_S is attained through the reaction of L-Cys with intracellular 3-mercaptopyruvate sulfurtransferase and cystathionine β-synthase in C6 cells. In addition to the incorporation of the substrate L-Cys, exogenous donors can be supplied to augment H_2_S production.^20^ Hu et al.^21^ designed a microneedle and loaded it with glucose oxidase and diallyl trisulfide for computed tomography imaging-guided gas starvation therapy combined with SDT/photothermal therapy for the treatment of breast tumors. Microneedle has the capacity to regulate the secretion of glucose oxidase and H_2_S, employing methodologies such as ultrasound or near-infrared laser irradiation to ensure a meticulous and controlled outcome. The experimental results demonstrated that photoacoustic stimulation in the presence of glutathione significantly augmented the efficiency of H_2_S production at pH = 5.4 (compared to pH = 7.4), thereby substantiating the pH responsiveness of H_2_S release. Li et al.^12^ prepared cascade bioreactors titanium sulfide nanosheets (TiSX NSs) for sequential gas-SDT. The release of gas H_2_S from TiSX NSs occurs independently of additional donors. After H_2_S generation, sulfur vacancies emerge on the surface of TiSX NSs, thereby enhancing the acoustic-dynamic properties. The experimental results also showed that H_2_S could inhibit the aggregation of myeloid-derived suppressor cells in the tumor cells, promote the proliferation of T cells inside the tumor, and the maturation of dendritic cells in the adjacent lymph nodes. These results indicate that H_2_S could activate the immune system of the body, thus enhancing therapeutic efficacy on the primary foci and metastatic foci.

### Carbon monoxide

**Figure 3:**
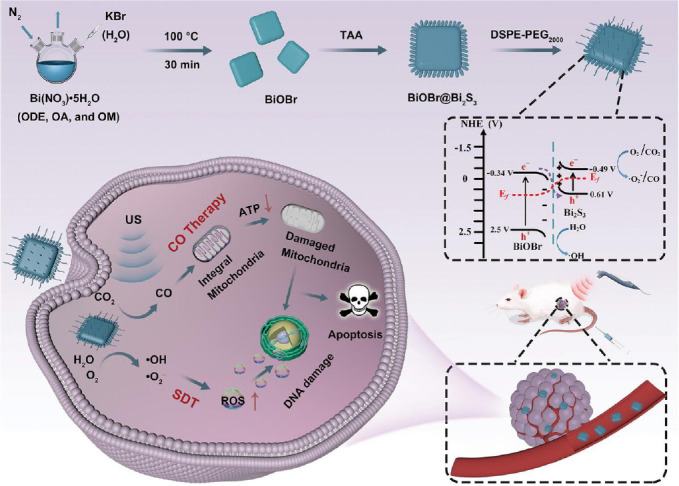
The synthesis and antitumor therapy of BiOBr@Bi_2_S_3_-DSPE-PEG2000. An interface‐engineered step‐scheme heterojunction based on BiOBr@Bi_2_S_3_ nanocomposite is developed to prolong the life span of sonoexcited electrons and holes, expand the selectivity of catalytic substrates and therapeutic products, and promote tumor catalytic therapy. Reprinted from Yuan et al.^25^ ATP: Adenosine triphosphate; DSPE: 1,2-distearoyl-sn-glycero-3-phosphorylethanolamine; PEG: polyethylene glycol; ROS: reactive oxygen species; SDT: sonodynamic therapy; TAA: thioacetamide; US: ultrasound.

In addition to NO and H_2_S, CO has been implicated in processes such as endogenous apoptosis and inflammatory responses. Moreover, CO stands as a widely studied gas signaling molecule. The administration of low doses of CO is believed to enhance the efficacy of autophagy inhibitors.^22^ Wang et al.^23^ developed a smart nanoplatform, the nanoplatform encapsulating mitoxantrone and manganese carbonyl (MCMA NPs), which is based on the chemotherapeutic drug mitoxantrone. MCMA NPs is designed for ultrasound/fluorescence imaging-guided chemotherapy-photothermal combination therapy. This pioneering study demonstrated the capacity of mitoxantrone to generate high heat under light for photothermal therapy, exhibiting a photothermal conversion efficiency of 42.2%. The study also confirmed that the sensitizing effect of CO chemotherapy enhanced the inhibitory effect of MCMA NPs on tumor growth. In addition to this, CO-releasing molecules are loaded into various nanocarriers and released upon ultrasound stimulation, a process that enables targeted delivery and controlled release of CO. A significant proportion of these CO-releasing molecules comprise metal carbonyl complexes, such as ruthenium carbonyls and manganese carbonyls. In the study by Yadav et al.^24^ a non-ferrocene analog [Mn(Ph-tpy)(CO)_3_ Br] Ph-tpy=4′-phenyl-2,2′:6′,2′′-terpyridine (Mn1) and a novel ferrocene conjugated Mn(I)-tricarbonyl complex viz [Mn(Fc-tpy)(CO)_3_Br] (Mn2), were prepared. The investigation revealed that Mn2 exhibited superior ultrasound-triggered ROS release properties in comparison to Mn1. A notable finding was the identification of a novel phenomenon wherein Mn(I) tricarbonyl complexes, Mn1 and Mn2, were found to be capable of producing ROS under ultrasound excitation. This release of ROS was further confirmed through the use of myoglobin assay and infrared spectroscopy, which provided evidence of ultrasound-activated properties in the complexes. The study demonstrated that both Mn1 and Mn2 exhibited significant necro-apoptotic cell death in both T-cell lymphoma and human breast cancer (MCF-7) cells. In contrast, Yuan et al.^25^ prepared a S-scheme heterojunction (BiOBr@Bi_2_S) by using the catalytic reduction of carbon dioxide (CO_2_) molecules to CO (**Figure 3**). Under ultrasound, H_2_O is oxidized to hydroxyl radical while oxygen and CO_2_ are reduced to 1O_2_ and CO, thus realizing the simultaneous combination of SDT and CO. This precise delivery of CO in the tumor area avoided the damage caused by CO in normal tissues and organs, producing a notable breakthrough in biosafety.

### Carbon dioxide

In addition to its function as a catalyst in the reduction of CO_2_ to CO, using specific materials, it has been demonstrated that CO_2_ itself functions as a signaling molecule that can play a role in tumor therapy.^26^ Chu et al.^27^ developed photoactivated contrast nano-agents to facilitate long-term ultrasound-guided combination therapies and to enable puncture biopsies for obtaining histologic specimens of lesions for precise pathological analysis. Compared to the clinically used contrast agent SonoVue (Bracco Diagnostics, Geneva, Switzerland), photoactivated contrast nano-agents demonstrate superior performance due to their capacity to generate gas continuously under 808 nm near-infrared laser irradiation. Consequently, this nano-diagnostic system holds considerable promise for clinical applications in ultrasound imaging-guided surgical treatment of tumors, including surgery, biopsy, and microwave ablation. In addition, the use of CO_2_ gas nuclei has been discovered to enhance the antitumor effects of SDT. Mo et al.^28^ developed a “lysosomal bomb” (PpIX-ACC@CMs) to target the lysosomes of tumor cells. The loaded amorphous calcium carbonate (ACC) acid reacted with the lysosomes, generating CO_2_ gas nuclei. This increased the sonication effect and exhibited lysosomal escape ability. *In vitro* cellular experimentation yielded results showing that cells treated in the PpIX-ACC@CMs group exhibited enhanced green fluorescence and elevated ROS levels in comparison to cells treated with PpIX@CMs. The underlying mechanism for this phenomenon may involve the degradation of PpIX-ACC@CMs in pathologically acidic tumor cells, which produces CO_2_, thereby promoting the cavitation effect of ultrasound. Furthermore, the results of the study suggest that Ca^2+^ overload contributes to the inhibition of tumor growth and the enhancement of computed tomography imaging for the purpose of monitoring efficacy.

### Oxygen

A prevailing challenge in the field is the understanding that oxygen deficiency frequently serves as the primary impediment to the effectiveness of tumor therapy. Two predominant oxygen delivery methodologies exist at present. One method involves the *in-situ* reaction of a metal or catalase loaded in a drug carrier with endogenous hydrogen peroxide from TME to generate oxygen. Yue et al.^29^ synthesized heterostructured manganese dioxide-coated BaTiO_3_ nanoparticles (BTO@M NPs) with the objective of achieving microenvironment remodeling by catalyzing the expression of hydrogen peroxide in TME. In this process, the NPs generate large amounts of oxygen while also consuming the antioxidant glutathione. However, this method of oxygen supply has limitations, resulting in suboptimal and less predictable outcomes. Another method of transporting oxygen is to deliver oxygen directly to the target area via drug carriers. This approach is undoubtedly the most direct and effective way to alleviate the hypoxic state of the TME. Wang et al.^30^ developed a Ce6-liposome-bound oxygen-carrying lipid microbubble microsystem. The findings from both *in vitro* and *ex vivo* investigations demonstrated that the combination of sonophotodynamic therapy and O_2_ gas therapy, mediated by lipo-Ce6, led to enhanced antitumor efficacy. Tan et al.^31^ developed oxygen-sufficient nanobubbles (IR780@O_2_ NBs) to mediate the enrichment and controlled release of NBs in the tumor region, providing more oxygen to the microenvironment to amplify the efficacy of SDT and break the barrier of nanoplatform delivery. The experimental findings disclosed that the local oxygen concentration attained levels 3-fold higher upon the integration of ultrasound and oxygen-carrying MBs. The IR780@O_2_ NBs with ultrasound-targeted microbubble destruction (UTMD) and ultrasound treated group exhibited the highest degree of ROS generation efficiency, suggesting that UTMD technology has the potential to directly mediate NBs, augmenting oxygen concentration. Furthermore, it was postulated that the augmentation of the number of pores and clathrin, as induced by UTMD, may have been a contributing factor to the observed increase in oxygen uptake. Moreover, increased oxygen levels have been evidenced to modify TME, consequently reducing the resistance of tumor tissue to other treatments, such as chemotherapy and radiotherapy.

### Other gases

SO_2_ has been documented to induce oxidative stress in biomolecules.^32^ Tian et al.^33^ designed a nanoreactor, CCM@FH-DNs, in which glutathione overexpressed in tumor cells triggered SO_2_ gas generation, enhancing SDT efficacy. It was demonstrated that the resultant SO_2_ could further inhibit superoxide dismutase and inactivate glutathione peroxidase 4. The extent of glutathione peroxidase 4 inactivation exhibited a positive correlation with the degree of oxidative stress within the tumor. Hydrogen (H_2_) is an emerging gas therapeutic molecule, and Qi et al.^34^ reported an H_2_-EC/SD therapy/imaging platform that produces H_2_ bubbles. These bubbles can be used both as contrast agents to enhance the imaging signal of tumors and to generate heat to damage tumor cells during ultrasound. Furthermore, H_2_ exerts a mechanically disruptive effect on the tumor vasculature and disturbs the homeostasis of TME. Manganese protocells (MnG) have demonstrated considerable promise as a novel gas delivery system. Prepared by liquid-phase stripping and *in situ* electrochemical replacement, MnG is capable of sustained generation of H_2_ and Mn^2+^ under physiological conditions. It has been demonstrated that MnG can not only activate the cyclic GMP-AMP synthase (cGAS)-stimulator of interferon genes (STING) pathway through the sustained release of Mn²^+^, but also synergistically enhance the activation of the cGAS-STING pathway by regulating tumor glucose metabolism and inhibiting the expression of three prime repair exonuclease 2. This mechanism enables MnG to significantly enhance the immunotherapeutic effect of hydrogen and convert “cold” tumors into “hot” tumors, thereby improving the response rate to immune checkpoint blockade therapy.^35^ In addition to the direct provision of specific gas signaling molecules, gas microbubbles can be generated under ultrasound amplitude illumination by providing gas precursors to enhance cavitation and thereby increase the efficiency of ROS generation. Lee et al.^36^ formulated nanoparticles (P-MSTNs) and encapsulated perfluorohexane (PFH) within the nanoparticles. The experimental results demonstrated that PFH@P-MSTNs could generate nanobubbles in the tumor region under low-frequency ultrasound stimulation, which resulted in the generation of significant amounts of ROS (a substantial enhancement in singlet oxygen sensor green fluorescence signal compared with P-MSTNs group). Combined studies of inert gas microbubbles and ultrasound have also been undertaken. *In vitro* experiments have demonstrated that the combined treatment reduced the proliferative activity of human pancreatic cancer PANC-1 cells and induced apoptosis. The cancer inhibitory effect of the joint treatment is obvious, and it provides a new combined strategy for SDT.^37^

**Figure 4:**
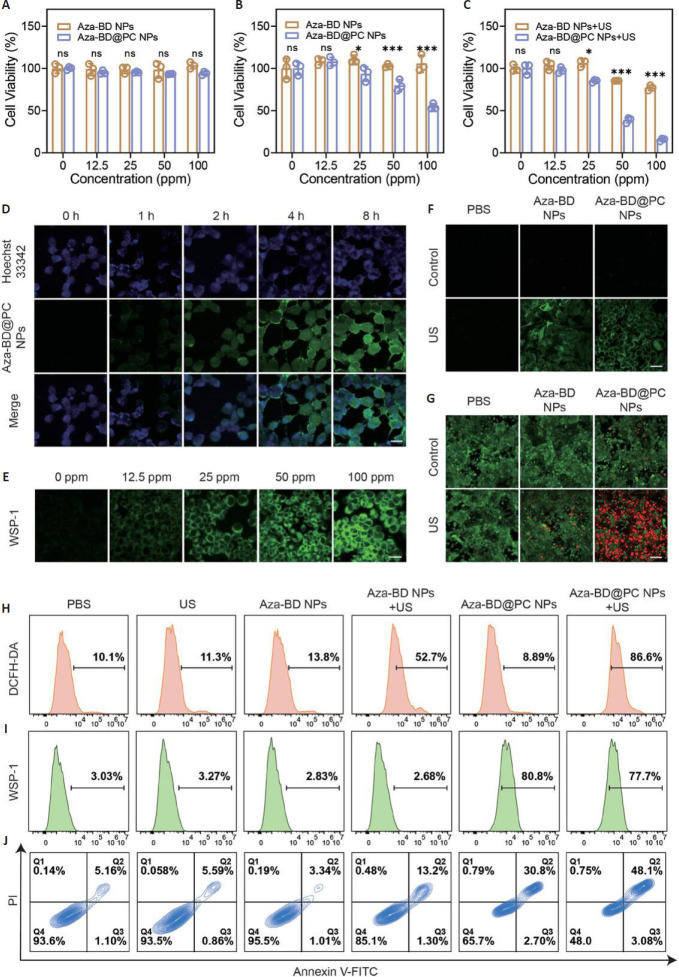
The efficacy of Aza-BD@PC NPs under ultrasound (US) exposure *in vitro*. (A, B) Cell viabilities of 3T3 cells (A) and GL261 cells (B) treated with different doses of Aza-BD NPs and Aza-BD@PC NPs (*n* = 5). (C) Survival rates of GL261 cells after treatment with different doses of Aza-BD@PC NPs under both US and non-US conditions (*n* = 5). (D) Confocal images of GL261 cells after incubation with FITC-labeled Aza-BD@PC-NPs for various incubation durations. Scale bar: 30 µm. (E) Confocal images of GL261 cells stained with WSP-1 after incubation with different concentrations of Aza-BD@PC NPs. Scale bar: 50 µm. (F, G) CLSM images of GL261 cells stained with DCFH-DA (F) and calcein-AM/PI (G) after various treatments. Scale bars: 50 µm for F and 100 µm for G, respectively. (H–J) Flow cytometry analysis of GL261 cells stained with DCFH-DA (H), WSP-1 (I), and Annexin V-FITC/P (J) I in diverse treated groups. Data are represented as mean ± SD and analyzed by one-way ANOVA. *P < 0.05, **P < 0.01, ***P < 0.001. Reprinted from Zhao et al.^47^ DCFH-DA: 2′,7′-Dichlorodihydrofluorescein diacetate; NPs: nanoparticles; ns: no statistical difference; PBS: phosphate buffered saline; WSP-1: Washington state probe-1.

It has been demonstrated that the combination of SDT with gas therapy has produced substantial synergistic anti-tumor effects in a variety of tumor types. In the case of solid tumors, such as those affecting the breast, lung, and liver, the combination therapy has been shown to significantly inhibit tumor growth and decrease metastasis by alleviating tumor hypoxia, enhancing the production of ROS, and inducing the apoptosis of tumor cells. Moreover, SDT combined with oxygen therapy has the capacity to penetrate the blood-brain barrier, thereby eliminating brain tumors, such as glioma cells.^38^ However, when applied to hematological tumors, such as lymphoma and pancreatic cancer, the combination therapy has shown limited efficacy, primarily due to the challenges associated with gas distribution and the dense nature of the stroma surrounding the tumor.^39^,^40^ A significant body of research has demonstrated a notable synergy between gas therapy and SDT, offering a promising avenue for enhancing cancer treatment outcomes (**Table 1**).^16^,^41^,^42^,^43^,^44^,^45^,^46^,^47^,^48^,^49^,^50^,^51^,^52^,^53^,^54^,^55^,^56^,^57^ This combined therapeutic strategy is capable of targeting the complexity and heterogeneity of tumors and acting on tumor cells through multiple mechanisms to achieve more effective tumor suppression. The gas therapy component serves to amplify the efficacy of SDT by providing oxygen and NO signaling molecules.^41^,^42^ These gaseous molecules have been observed to enhance ROS generation and alleviate tumor hypoxia. Furthermore, the combination of gas therapy and SDT can facilitate tumor-targeted delivery of gaseous molecules through stimuli-responsive nanocarriers, which can enhance the precision and efficiency of treatment (**Figure 4**).^47^ The combined application of SDT and gas therapy has opened new avenues for tumor treatment, and the key role of aerodynamics in this combined strategy cannot be ignored. By precisely regulating the transport and distribution of gases in tumor tissues, aerodynamics not only optimizes the delivery efficiency of gas molecules but also significantly enhances their therapeutic effects. For example, in combination with nanotechnology and biomaterials, aerodynamics enables the design of smarter gas delivery systems for precise regulation of TME.^12^ Consequently, there is a need for further exploration into innovative applications of gas kinetics in the combination therapy, particularly in the domain of gas delivery vehicle development, such as responsive nanoparticles or microbubbles, aimed at enhancing the enrichment and release efficiency of gas molecules at the tumor site. Additionally, there is a necessity for in-depth investigation of the interaction mechanism between aerodynamics and TME. This investigation should prioritize elucidation of its potential role in regulating immune response and inhibiting tumor metastasis. In conclusion, aerodynamics provides a significant technical framework and theoretical foundation for the combined application of SDT and gas therapy. Through multidisciplinary collaboration and technological innovation, this combined strategy is expected to generate breakthroughs in tumor therapy, providing patients with more efficient and precise treatment options.

**Table 1:** Various types of sonosensitizers for gas-combined sonodynamic therapy (SDT) in cancer treatment

Gas	Sensitizer	Design principle of sensitizer	Combined treatment	Tumor model	Reference
NO	QD@Ca/ML-Arg	A CaCO_3_ nanoplatform encapsulated Ag_2_S and loaded with L-Arg and further wrapped with red blood cell/platelet membrane	SDT Ca^2+^	Breast cancer	41
	T-mTNPs@L-Arg	TPP was modified on the surface of L-Arg loaded mTNPs for mitochondria-targeting	SDT	Breast cancer	42
	BP@Cu_2_O@L-Arg	Cu_2_O particles were generated *in situ* on the surface of the BP nanosheets to harvest breast cancer and incorporated L-Arg into breast cancer through electrostatic interactions	SDT CDT IT	Breast cancer	43
	Arg@VMT@PDA	Constructed on 2D porous vermiculite nanosheets adsorbed L-Arg, and the nanosheets' surface was coated with polydopamine and polyethylene glycol	SDT CDT	Breast cancer Liver cancer	16
	BMT@LA NCs	A US-triggered "nanovaccine" like system based on L-Arg-loaded black mesoporous titania nanocomposites	SDT IT	Cervical cancer	44
	^131^I-SD-MnO_2_-HA	SD was synthesized to release NO, and ^131^I and HA-modified MnO_2_ nanocarrier (MnO_2_-HA) was chosen as an encapsulating matrix	SDT CDT RIT	Cervical cancer	45
	BST@LA	Sr-doped and oxygen vacancy defect-engineered BaTiO_3_ piezoelectric nanoparticle loading with L-Arg	SDT	Breast cancer	46
H_2_S	Aza-BD@PC	An intelligent nanoplatform based on the Aza-boron-dipyrromethene dye and phenyl chlorothionocarbonate-modified DSPE-PEG molecules	SDT	Gliomas	47
	SPNDNH	A semiconducting polymer, H_2_S donor, and indoleamine 2,3-dioxygenase inhibitor (NLG919) are encapsulated by singlet oxygen-responsive shells with modification of hyaluronidase	SDT IT		48
CO	Re-Cy	Cyanine moieties were appended in the complex and increase its sonosensitivity for CO release	SDT	Breast cancer	49
	PM-FM/C	Macrophage membrane inserted with SP94-peptide was cloaked on FM/C to construct a dual-targeting biomimetic nanoplatform	SDT CDT	Liver cancer	50
CO_2_	iCRET	Chemiluminescence resonance energy transfer-based immunostimulatory nanoparticles	SDT ICB	Colorectal cancer	51
	Ca@H	Hematoporphyrin monomethyl ether loaded CaCO_3_ nanoparticles were synthesized by a gas diffusion method	SDT	Breast cancer	52
	CTC	pH-dissociable CaCO_3_ nanoparticles, as a type of calcium ionophores, were selected as the depot for the encapsulation of CAR and meso-tetra-(4-carboxyphenyl)porphine to formulate CTC as an ion homeostasis perturbator	SDT IIT	Breast cancer	53
SO_2_	PBSA-EG	A multicomponent polymerization approach involving bipyrroles, sulfonyl azides, and diynes was developed to afford a library of PPSIs in high yields and molecular weights, which were further modified to form unique SO_2_ generators	SDT	Liver cancer Cervical cancer	54
	Aza-DNBS	Self-assembling Aza-boron-dipyrromethene based sonosensitizer molecules and 2,4-dinitrobenzenesulfonate-caged SO_2_ prodrug	SDT ICB	Liver cancer Lung cancer	55
	P-DOA	A dual-prodrug molecule of SO_2_ and 5-ALA is synthesized and co-assembled with methoxyl poly(ethylene glycol)-b-poly(l-lysine) to generate the two-in-one prodrug nanoparticles	SDT	Melanoma squamous cell carcinoma	56
H_2_	Pt-Bi_2_S_2_	The narrow-bandgap semiconductor bismuth sulfide is selected as the sonocatalyst and platinum nanoparticles are grown *in situ*	SDT	Breast cancer	57

^131^I: 131-iodine; 2D: two-dimensional; 5-ALA: 5-aminolevulinic acid; Ag_2_S: silver sulfide; BaTiO_3_: barium titanate; BP: black phosphorus; Ca^2+^: Ca^2+^ interference therapy; CaCO_2_: calcium carbonate; CAR: carvacrol; CDT: chemical dynamic therapy; CO: carbon monoxide; CO_2_: carbon dioxide; CTC: CaCO_3_@TCPP/CAR; Cu2O: cuprous oxide; DSPE-PEG: 1,2-distearoyl-sn-glycero-3-phosphorylethanolamine-polyethylene glycol; FM/C: a muti-modal therapeutic nanoplatform; H_2_: hydrogen; H_2_S: hydrogen sulfide; HA: hyaluronic acid; ICB: immune checkpoint blockade; IIT: interference therapy; IT: immunotherapy; L-Arg: L-arginine; MnO_2_: manganese dioxide; mTNPs: mesoporous titanium dioxide nanoparticle; NO: nitric oxide; PPSI: poly(bipyrrole-sulfonylimide); RIT: radiation therapy; SD: a bifunctional small-molecule donor; SDT: sonodynamic therapy; SO_2_: sulfur dioxide; Sr: strontium; TCPP: an ion homeostasis perturbator; TPP: triphenyl phosphonium; US: ultrasound.

## Other Combined Strategies with Sonodynamic Therapy in Tumor Treatment

Photodynamic therapy (PDT) has demonstrated significant efficacy in the treatment of superficial skin, esophageal, and lung cancers. Numerous clinical trials have validated the potential of PDT for the treatment of other tumor types.^58^ However, the scattering and absorption of light in tissues can hinder the efficacy of PDT in treating deep-seated tumors. Conversely, the ultrasound applied in SDT exhibits superior tissue penetration, thereby significantly amplifying the therapeutic outcome. The application of suitable sensitizers has been shown to generate ROS under conditions of light and ultrasound, thereby enhancing ROS generation efficiency, tissue penetration capability, and tumor targeting.^59^

Immunotherapy, a type of treatment, is defined as the process by which the immune system is stimulated to combat tumor cells.^60^,^61^ SDT employs ultrasound to trigger the activation of acoustic sensitizers, which in turn generate ROS. This process ultimately results in the immunogenic death of tumor cells, thereby inducing tumor-specific immune responses. The combinatorial approach of SDT and immunotherapy has been shown to produce synergistic anti-tumor immune effects. Immunogenic cell death is triggered during SDT, and dying tumor cells release signaling molecules that can stimulate anti-tumor immune responses *in vivo*, collectively known as damage-associated molecular patterns.^62^ These DAMPs are recognized by intrinsic pattern recognition receptors, such as Toll-like receptors and NOD-like receptors, which promote the maturation of DCs and activate CD8^+^ T cell immune responses.^63^

## Conclusion

This review serves as a reference for the further application of sonodynamic combination therapy by providing an overview of the progress in research related to the use of SDT in conjunction with gas therapy and other therapeutic modalities, particularly for the treatment of tumors. The combination of SDT with these other therapeutic interventions has also demonstrated significant potential for application. It is recommended that subsequent studies focus on the exploration of these combined therapeutic strategies to enhance the clinical application of SDT. Nevertheless, despite the advances in SDT combination therapy, there are several issues that still require resolution. Initially, the development of novel targeted acoustic sensitizers, including tumor-specific antigen-targeting, receptor-targeting, gene-targeting, and other approaches, holds promise in enhancing the diagnostic accuracy and therapeutic efficacy of SDT. Thirdly, notwithstanding the encouraging applications of SDT in animal model studies, further exploration is required regarding its current application and potential clinical translation in human tissues. Secondly, at present, the spatial and temporal heterogeneity of SDT efficacy remains to be studied. Furthermore, the question of whether SDT exerts the same effect in primary and metastatic foci requires further validation. In the context of specific tumor subtypes, SDT has the potential to function as a targeted therapeutic agent when employed in conjunction with gene therapy.^56^ In the future, the killing effect of SDT on heterogeneous tumors needs to be further improved by optimizing the design of acoustic sensitizers, the combined treatment strategy, and the precise implementation, which will provide new ideas for clinical translation.

The combined use of SDT can also result in adverse effects. The combination of SDT and gas therapy has been observed to enhance the production of ROS, which, in excessive amounts, can induce oxidative stress damage in normal tissues. The diffusion of gases, such as SO_2_ and H_2_S, during gas therapy, can reach non-targeted regions, resulting in non-specific toxicity. Phototoxicity and mechanical damage caused by sonophotodynamic therapy may lead to inflammatory reactions, such as erythema, pruritus, and skin or mucous membrane erosion.^64^ Additionally, local tissue necrosis or damage during treatment can elevate the risk of infection. The combination of SDT and immunotherapy has been demonstrated to elicit a dual effect on the immune system. On one hand, TME may be over-suppressed by an activated immune response, resulting in a poor therapeutic outcome. On the other hand, immune over-activation may lead to an autoimmune response that attacks normal tissues.^55^,^65^

Consequently, the diagnostic and therapeutic approaches employed in the management of tumors are evolving towards enhanced precision and effectiveness. The ongoing investigation and implementation of pioneering therapeutic modalities, including SDT, gas therapy, PDT and immunotherapy, have culminated in a proliferation of treatment alternatives for patients afflicted with tumors. This article is a retrospective literature review. Despite the utilization of a set of screening criteria for the evaluation of literature selection, a paucity of consensus persists regarding unified standards for data acquisition and analysis. This absence of consensus may result in the presence of certain biases in the results. The heterogeneity of experimental models (e.g., cell lines, animal species) and therapeutic parameters (e.g., ultrasound intensity, gas dosage) across the included studies complicates the direct comparability of outcomes. In the future, as research progresses in the direction of a more profound comprehension of molecular mechanisms and the development of novel therapeutic strategies, there is an expectation of further improvements in prognoses for tumor patients.

## Data Availability

*Not available*.
